# Translation, transcultural adaptation into Brazilian Portuguese and concurrent validity of the rheumatoid arthritis assessment scale (RAKAS–13/Brazil)

**DOI:** 10.1186/s42358-023-00341-z

**Published:** 2024-01-02

**Authors:** Lindomar Mineiro, Tamires Terezinha Gallo da Silva, Silvia Regina Valderramas, Sergio Candido Kowalski, Eduardo dos Santos Paiva, Anna Raquel Silveira Gomes

**Affiliations:** 1 Physical Education Program, Federal University of Paraná, Curitiba, Paraná Brazil; 2 Internal Medicine and Health Sciences, Department of Prevention and Rehabilitation in Physical Therapy, Federal University of Paraná, Curitiba, Paraná Brazil; 3 UFPR, Faculty of Health Sciences of HEI, McMaster University, Hamilton, Canada; 4 Rheumatology, Internal Medicine Department, Federal University of Paraná, Curitiba, Paraná Brazil; 5 Prevention and Rehabilitation in Physical Therapy Department, Masters and PhD Programs in Physical Education, Federal University of Paraná, Curitiba, Paraná Brazil

**Keywords:** Rheumatoid arthritis, Patient education, Self care, Rheumatic Disease, Patient Health Questionnaire

## Abstract

**Background:**

Knowledge of patients about Rheumatoid Arthritis (RA) is a necessary aspect to better approach self-management support in a patient-centered manner. The research instrument known as the Rheumatoid Arthritis Knowledge Assessment Scale (RAKAS), consisting of 13 items, is simple, reliable and reproducible, and can be applied in both clinical practice and research protocols.

**Objective:**

This study aimed to translate and culturally adapt the RAKAS vocabulary into Brazilian Portuguese and to evaluate its concurrent validity.

**Methods:**

The RAKAS was translated into Brazilian Portuguese and administered to 52 elderly women with RA recruited between May 2021 and May 2022. Concurrent validity was assessed using the Spearman’s correlation coefficient between RAKAS and Patient Knowledge Questionnaire (PKQ).

**Results:**

The participants considered RAKAS-13/BRAZIL easy to understand and did not report any doubts in answering the final version. Concurrent validity of the RAKAS–13/BRAZIL was low compared to the PKQ (*ρ* = 0.283, *p* = 0.038). Conclusion: The Brazilian Portuguese version of the RAKAS (RAKAS–13/BRASIL) proved to be a questionnaire that was easy and quick to administer to assess patient knowledge about Rheumatoid Arthritis, despite its low correlation with the PKQ in the present study.

**Supplementary Information:**

The online version contains supplementary material available at 10.1186/s42358-023-00341-z.

## Background

Rheumatoid Arthritis (RA) is a chronic, progressive, disabling disease with a profound impact on daily self-care [1, 2]. RA treatment goes beyond drug therapy and involves the ability to understand the disease and deal with the practical, physical and psychological impacts that come along with it. Patients’ knowledge about RA can contribute to better awareness and self-management of the disease, which is crucial to reduce disability [3].

It appears that patient knowledge is an effective indicator of self-care and home management of RA related complications [3]. However, the low educational level of RA patients can impair early diagnosis [4]; increase joint damage and is directly linked to low adherence to pharmacological and non-pharmacological treatments [5].

The Brazilian Ministry of Health, through the protocol of therapeutic guidelines for RA, suggests the inclusion of patient education protocols to contribute to the treatment of RA [6, 7]. In this context, the Brazilian Society of Rheumatology supports the adaptation of questionnaires to assess patient knowledge, as they are items of great importance for the provision of adequate instruments for conducting clinical protocols [8]. In this way, proposals for cross-cultural adaptation and reliability of existing questionnaires are being encouraged, as they can contribute to normalize and standardize international technical language [9].

In Brazil there is only one tool designed to assess patient’s knowledge about RA, called Brazil Patient Knowledge Questionnaire - PKQ [10]. The authors of PKQ concluded that patients with RA had low knowledge about RA, and patients with less time of formal study and older had lower scores of knowledge, in other words, elderly people with low educational level had lower scores of knowledge about the illness. This may be related to the difficulty in understanding the questionnaire, according to the authors [10].

Therefore, in Brazil, there is still a need for a tool/questionnaire that may be easy to apply and allow satisfactory findings about all levels of schooling, as well as elderly patients. Naqvi et al. [2], with the intention of investigate the knowledge of patients affected by RA, developed the Rheumatoid Arthritis Knowledge Assessment Scale (RAKAS) questionnaire, an instrument that allows patients the assessment of knowledge through items of easy application about Rheumatoid Arthritis disease, its risk factors, symptoms and treatment [2].

The RAKAS has not been translated yet and cross-culturally validated into Brazilian Portuguese, which makes its application unfeasible.

Considering the need for assessment of the older Brazilians patients’ knowledge regarding RA, and the lack of instruments in Brazilian Portuguese, the objective of the present study was to translate and cross-culturally adapt the RAKAS–13/BRAZIL (Escala de Avaliação de Conhecimento sobre Artrite Reumatoide) into Brazilian Portuguese and to evaluate its concurrent validity.

## Methods

### Design

Cross-sectional study.

### Ethical aspects

This is a cross-sectional study of the translation and cross-cultural adaptation and was approved by the Research Ethics Committee of the Complex Clinic´s Hospital of the Federal University of Paraná, Brazil (CHC/UFPR) (CAAE 29628119.5.0000.0096; register 3.951.778), according to National Health Council Resolution 466/2012.

Prior consent from the creator of the *Rheumatoid Arthritis Knowledge Assessment Scale* (RAKAS) was obtained from Dr. Atta Abbas Naqvi, at the University of Universiti Sains Malaysia, School of Pharmaceutical Sciences Malaysia, Penang.

#### Participants

The study population consisted of elderly women with Rheumatoid Arthritis (RA), living in the community. The inclusion criteria were older women with RA (65 years or older), who were seen in an outpatient Rheumatology’s unit of the CHC/UFPR, in the city of Curitiba, between May 2021 and May 2022. The medical records available at the rheumatoid arthritis’s outpatient clinics from the CHC/UFPR were analyzed, considering the criteria for the records selection: patient at the reception waiting or during the medical assessment; patient residing at the city of Curitiba; patient able to perform the assessments considering motor and cognitive skills; agree to participate of the study. The present study was conducted on convenience and purposive sample, no randomly, assessing only women over the age of 65 and not included young women and men because RA is more prevalent in women and its related complications are worst in older ones [11, 12].

The patients were diagnosed with RA according to American College of Rheumatology (ACR) 1987 and 2010 ACR and European League Against Rheumatism criteria [13]. The participants were directly invited to participate in the study by one of the researchers while waiting for a visit with a rheumatologist. Those who agreed to participate in the research signed the Free and Informed Consent Form. Individuals who scored less than 18/19 (for participants with no formal education) and less than 24/25 (with formal education) on the Mini-Mental State Examination (MMSE) were excluded for possible cognitive impairment [14]. The exclusion criteria were neurological disorders and/or traumatic-orthopedic conditions that prevented the participant from carrying out the evaluations.

Two physical education professionals and one physical therapist collected the data at the clinic. The determination of the sample size occurred as proposed by Terwee et al. [15], which proposes from 4 (minimum) to 10 (maximum) participants for each question of the instrument to be translated. The RAKAS has 13 questions, so a minimum of 52 participants were needed in this study [15].

### Measures

#### Demographics and clinical characteristics

##### Body mass index (BMI)

The participants’ body mass and height were measured using a Balmak® mechanic anthropometric scale. Their BMI was classified considering the following thresholds: underweight (BMI < 23 kg/m2), normal (23 < BMI < 28 kg/m2), overweight (28 < BMI < 30 kg/m2), obesity (BMI > 30 kg/m2)[16].

##### C-reactive protein (CRP) and erythrocyte sedimentation rate (ESR)

The CRP and ESR were used to assess inflammatory RA activity. For this study it was considered values of ESR > 28 mm/h and CRP 10 mg/L [17, 18].

##### Disease activity score (DAS28)

To assess disease activity, the DAS28 was used [19]. ESR and CRP can be used to measure the degree of inflammation in the serum, usually with one of the readings, ESR or CRP [20] being used to calculate the DAS28 for the patient. Pain was quantified through the global assessment of the disease assessed using the visual analogue scale (VAS), from zero (no pain) to 100 mm (very intense pain) [21]. RA remission was considered when the DAS28 score was less than 2.6; low activity from 2.6 to 3.2 points; moderate activity from 3.2 to 5.1 and intense activity above 5.1 points, with 10 being the maximum index score [19]. The application and calculation of the DAS28 was performed by rheumatologists from the Rheumatoid Arthritis clinic. The researchers of the present study collected the DAS28 result by consulting the medical records, during or after the patient’s consultation with the rheumatologist.

##### Health assessment questionnaire (HAQ)

To assess the functional capacity of the participants, the HAQ was used [22, 23]. The result of the RA severity level was given as a result of the total average being classified as without disability (HAQ 0); Mild deficiency (HAQ 0–1); Moderate deficiency (HAQ 1–2); Severe deficiency (HAQ 2–3) [22, 23].

### Demographic characteristics

A sociodemographic assessment was carried out by an interviewer for all the participants about: race; level of education; marital status; occupation; residence; income; dominant side and disease duration.

#### Rheumatoid arthritis knowledge assessment scale (RAKAS)

The RAKAS questionnaire was designed to document the patient’s knowledge of RA. The questionnaire has 13 multiple choice questions. One question (question 01) has two correct answers and the others, only one correct answer, and two to four distractors [2].

The patient’s knowledge about the disease, its risk factors, symptoms and treatment are categorized into four levels: excellent knowledge (11–14 points - >75% of the questions); adequate (8–10 points − 50 − 75% of the questions); low (5–7 points − 30 − 50% of the questions) and bad (≤ 4 points - < 30% of the questions) [2].

#### Brazil patient knowledge questionnaire (PKQ)

The PKQ was developed by Hennell et al. [24] in 2004, in the United Kingdom, and translated and adapted into Brazilian Portuguese by Jennings et al. [10] in 2006. The PKQ is an instrument translated and validated into Brazilian Portuguese to assess the knowledge of patients with RA. It has 16 questions, some of which may have more than one correct answer, with a maximum possible score of 30 points, adding all the correct answers [10].

### Translation, cross-cultural adaptation

**Fig. 1:**
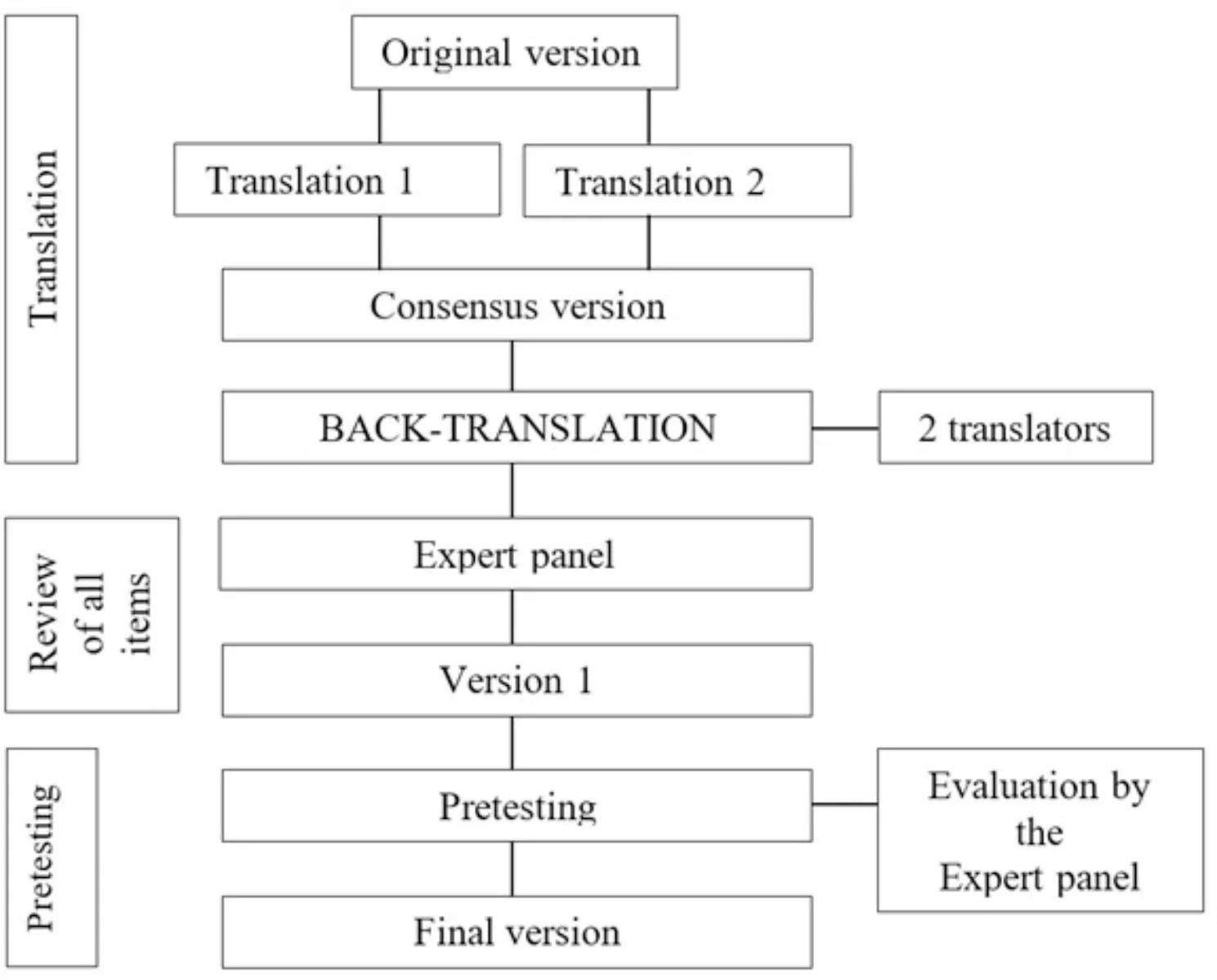
Flowchart showing the stages of translation and cross-cultural adaptation of the RAKAS questionnaire

The translation and cross-cultural adaptation of RAKAS was performed according to Beaton et al. [25], following the steps: (1) Translation, (2) Synthesis, (3) Retro translation, (4) Expert committee (review and pre-final version), (5) Pre-test, (6) Expert committee analysis and creation of the final version of the instrument (Fig. 1).

Two bilingual (Portuguese/English) translators (SK and VT), native from Brazil, fluent, independent, performed the translation of the questionnaire into Brazilian Portuguese, one of the translators being a professional in the health area (SK), with prior knowledge of the objectives of the study, and the other, an English language teacher (VT), without prior knowledge of the study. From this stage onwards, two initial translations into Portuguese emerged, version 1 (T1) and version 2 (T2).

1. The two translations were compared and analyzed in a meeting with the translators and researchers involved in the study. From the two initial translations, differences were reduced, preserving the cultural context of the Brazilian population and the original concepts of the instrument, thus, a consensual version of the questionnaire in Portuguese was obtained, called T12.
2. Two other English teachers (AF and RG), bilingual, being native English speakers, independent and qualified, did the back translation, that is, from Brazilian Portuguese version into English. The translators at this stage did not receive any information about the study or the original questionnaire.
3. The T12, back translation and original versions were submitted to a committee of experts, composed of four bilingual translators (SK; VT; AF and RG), together with 04 health professionals (01 Physical Educator - LM, 02 Physical Therapists – ARSG and SRV and 01 Rheumatologist - SK). The experts evaluated semantics, idiomatic expressions, cultural and conceptual equivalences, and identified and discussed possible discrepancies. After consensus, a new Portuguese version of RAKAS was established (RAKAS − 13/BRAZIL version 1, with its respective English version).
4. In the pre-test phase, evaluator 1 (LM) applied the RAKAS − 13/BRAZIL version 1 to elderly women (n = 18) with RA attending the rheumatology outpatient clinic at CHC/UFPR. All questions were fully understood by the participants and the evaluator had no difficulty in applying them.
5. Finally, an analysis was carried out by the committee to discuss the results of the pre-test and obtain the final version of RAKAS − 13/BRAZIL.

### Concurrent validity

The concurrent validity [26] of the RAKAS-13/Brazil questionnaire was tested to determine its relationship with the questionnaire PKQ. The PKQ is a questionnaire that has a similar concurrent validity to the RAKAS − 13/BRAZIL.

### Statistical analysis

The Statistical Package for the Social Sciences (SPSS) software, version 21 was used to perform the statistical analysis.

The results are presented in descriptive statistics (mean ± standard deviation, absolute and relative frequency). To analyze the concurrent validity, Spearman’s correlation coefficient was used between the RAKAS–13/BRAZIL and PKQ-Brazil instruments. The magnitude scale proposed by Hopkins was used to interpret the correlation coefficients, being: < 0.1, trivial; between 0.1 and 0.29, small; 0.30–0.49, moderate; 0.50–0.69, high; 0.70–0.90, very high; >0.90, almost perfect [27].

## Results

Medical records available at the rheumatoid arthritis’s outpatient clinics from the Complex Clinic’s Hospital of the Federal University of Paraná were analyzed and 79 medical records from elderly women with RA could be assessed for the study. However, 15 older adults’ women left the local before being asked to participate; 6 were excluded (1 did not live in Curitiba; 3 used wheelchair and 2 did not reach the minimal score in the MMSE); 6 refused to participate (4 did not have time enough; 1 because was hungry and 1 was worried about COVID19 contamination). A total of 52 older adults women with RA (mean 71.2 ± SD 5.4 years old) were included.

The clinical and demographic characteristics are shown in Table 1.

The RAKAS–13/BRAZIL score, for this study, showed that 30.77% of participants had excellent knowledge about RA; 51.92% adequate knowledge and only 17.31% presented low knowledge. None of them presented poor knowledge.

The RAKAS mean score of the sample was 9.7 meaning adequate knowledge about Rheumatoid Arthritis (RA), with mean response time of 3.2 ± 0.43 min. With the PKQ, 42% of the participants got 12.6 questions right out of 30, with an average response time of 9.38 ± 2.35 min (Table 2).

The concurrent validity between the RAKAS–13/BRAZIL and the PKQ - Brazil was small (ρ = 0.283, ρ = 0.038).

**Table 1:** Demographics and clinical characteristics of the participants (n = 52)

CHARACTERISTICS		Mean	SD	n	%
AGE (years)		71.2	5.4		
BMI (kg/m²)		28.2	5.4		
UNDERWEIGHT				8	15.38
NORMAL WEIGHT				19	36.54
OVERWEIGHT				5	9.62
OBESITY				20	38.46
MMSE		25.3	3.8		
HAQ		1.8	1.4		
ESR (mm/h)		40.2	21.0		
CRP (mm/L)		1.2	2.1		
DAS-28		3.2	1.3		
RACE	White			20	33.3
	black			4	6.8
	brow			9	15.3
EDUCATION	no schooling			42	71.2
	Incomplete Elementary School (1–3 years of schooling)			3	5.1
	Complete Elementary School (4 years of schooling)			0	0.0
	Incomplete High School (5 to 10 years of schooling)			4	6.8
	Complete High School (≥ 11 years of schooling)			0	0.0
	Incomplete Higher Education (≥ 14 years of schooling)			3	5.1
	Complete Higher Education (≥ 15 years of schooling)			0	0.0
	Master’s or Doctorate			42	71.2
MARITAL STATUS	Single			5	8.5
	Married			12	20.3
	Divorced c			9	15.3
	Widow			16	27.1
OCCUPATION	Employee			0	0.0
	unemployed			4	6.8
	retired			36	61.0
	Domestic			2	3.4
	self-employment/private			42	71.2
RESIDENCE	urban			51	98.08
	Rural			1	1.02
MONTHLY FAMILY INCOME	≤R$ 1.212.00 1 i.e. USD 227.31			9	15.3
	R$ 1.212.00 a R$ 4.848.00 i.e. USD 227.31-909.24			27	45.8
	R$ 4.848.00 a R$12.120.00 i.e. USD 909.24–2.273.1			4	6.8
	R$12.120.00 a R$24.240.00 i.e. USD 2.273.1-4.546.2			0	0.0
	≥R$24.240.00 i.e. over USD 4.546.2			0	0.0
DOMINANT SIDE	Right			51	98.08
	Left			1	1.02
DISEASE DURATION	< 10 years			4	7.69
	≥ 10 years			48	92.31

BMI, body mass index; Mini-Mental State Examination (MMSE); HAQ, Health Assessment Questionnaire; ESR, Erythrocyte Sedimentation Rate; CRP, C-Reactive Protein; DAS-28, Disease Activity Score; RAKAS, Rheumatoid Arthritis Knowledge Assessment Scale; PKQ, Brazil Patient Knowledge Questionnaire. N, number of participants; SD, standard deviation; Kg, kilogram; m², square meter; mm/h, millimeters per hour; mm/L, millimeters per liter; %, percentage. Note: 1 USD equals 5.32 R$ reais (Brazilian currency) at the time of this manuscript was written

During the translation process into Brazilian Portuguese, the two forward translations presented some differences. The committee reached a consensus for determining different choices of words, without changing the meaning of the sentence (Additional file 1). The same happened in the back-translation from Brazilian Portuguese into English (Additional file 2).

The pre-final version of the RAKAS − 13/BRASIL was applied to 18 elderly people in the form of an interview. Each question was followed by the item “did you understand this question?” All the participants, i.e., 18 older adults’ women with RA understood 100% of the questions. Thus, the version of the RAKAS − 13/BRAZIL was not modified, being applied in more 34 participants, concluding the adaptation process.

**Table 2:** The RAKAS-13/BRAZIL and PKQ-Brazil data of the participants (n = 52)

CHARACTERISTICS	RAKAS–13/BRAZIL (Mean ± SD)	PKQ-Brazil (Mean ± SD)
SCORE OF CORRECT ANSWERS (POINTS)	9.7 ± 2.0	12.6 ± 3.5
TIME TO APPLY (MINUTES: SECONDS)	3:2 ± 0.43	9:38 ± 2.35

SD, standard deviation

## Discussion

The version of the RAKAS-13 was translated and adapted transculturally into Brazilian Portuguese. The RAKAS–13/BRAZIL was a useful instrument to assess knowledge of a sample of elderly Brazilian patients about Rheumatoid Arthritis. The translation, synthesis and back translation steps of the RAKAS–13/BRAZIL were straightforward since there were not many differences between translated terms. The most part of the older adults’ women showed adequate knowledge about RA with the RAKAS–13/BRAZIL.

The acceptance of the RAKAS–13/BRAZIL from the participants of the present study was 100%, being higher than that reported by the original version of the RAKAS, 89.6% [2]. We can highlight the easiness that patients showed to understand the RAKAS–13/BRAZIL. When used the Brazilian version of PKQ, the patients needed help during its application, according to Jennings et al. [10], a low level of education was found in the sample with RA, many of whom illiterate, and the PKQ was reported as difficult for patients due to its structure. This fact, according to the authors, is due to the low level of education found in the Brazilian population with RA [10]. These findings are confirmed by other studies [2, 10, 24, 28, 29], which concluded that the level of education can interfere with the understanding of patients with lower scores when answering questionnaires concerning knowledge about RA.

However, in the present study, all the sample was older women and 71.2% of the sample had only 1 to 3 years of formal schooling and 3.4% were illiterate, different from the studies cited above, whereupon 51.92% showed adequate knowledge about RA. Even so, the RAKAS − 13/BRAZIL, showed excellent knowledge, 30.77%, about RA with a high mean response rate equal to 9.7 ± 2.0 correct of 14 answers. In the original version of RAKAS, in Pakistani, more than 70% of the participants were adults and being 47.5% graduated, it was found a mean response rate equal to 8 from 14 [2]. In the English version of RAKAS 75% of patients were adults and 59.9% declared graduated; 10.7% post graduated presenting excellent level of knowing about RA, i.e., a mean response rate equal to 11 from 14 answers [28].

In a study that compared the knowledge of patients about RA in Germany using the PKQ, it was found adequate knowledge about RA, even with a low educational level (4 years), suggesting that the level of schooling and the time of disease diagnosis might not impair the knowledge of patients about RA [30]. In the other hand, another study, carried out in the city of Curitiba/PR, the same city where the RAKAS-13/BRAZIL application took place, Souza Filho et al. [31], investigated the level of literacy from 72 individuals aged up to 65 years old, diseases not specified, comparing older people that completed higher education with others with less time of formal schooling. They found that more time in formal schooling did not determine better comprehension when compared with minor time of formal schooling.

The average RAKAS-13/BRASIL scores (9.7 ± 2.0 of 14 questions) outcomes from the present study are higher than those reported in the Pakistani version (7.63 ± 2.9 of 14 questions) and the English version (7.68 ± 2.52) of the RAKAS even with patients presenting more than 6 years of formal education [2, 28].

In the present study, data from the application of the PKQ showed an average of 12.6 ± 3.5 correct answers, similar to the study by Jennings et al. [10], with 12.96 ± 4.37. It also corroborates with the study by Gomes et al. [32], who, applying the PKQ, obtained a score of 13 ± 3.65 correct answers out of a maximum of 30. That is, less than half of the questions were answered correctly, which may be linked to the difficulty of understanding, even when applied by an interviewer [10; 24]. Moreover, after 2004, at least 8 new biological drugs were included in the treatment of RA, which has made it difficult to use the PKQ, as it induces the patient to error on questions related to drug knowledge [33, 34].

In the original English version or in the Brazilian Portuguese version [10], the PKQ does not present a way of classifying the patient’s level of knowledge through the score obtained. In this way, it is not possible to compare the level of knowledge between studies, since the studies that used the PKQ do not show, for example, whether the level of knowledge was high, medium, or low. This is an important gap observed, as it makes it difficult for health professionals to provide feedback to the patients and to compare studies carried out with the PKQ by the global community.

However, considering the time we have taken to apply RAKAS − 13/BRAZIL, 3:2 ± 0.43 min and the time to carry out PKQ – Brazil, 9:38 ± 2.35 min, we can note that the time we took to apply PKQ was less than the time reported in the translated version to Brazilian Portuguese, i.e., 10:31 ± 1.9 min [10]. Even so, RAKAS–13/BRAZIL application was 65.67% faster when compared to PKQ - Brazil.

Nevertheless, it was found a small concurrent validity between the RAKAS–13/BRAZIL and the PKQ – Brazil, probably due to formentioned reasons. The PKQ was chosen because it is the only questionnaire known so far to analyze the knowledge of patients with RA, translated and validated into Brazilian Portuguese [10].

The present study has some limitations, such as the use of only one instrument to test the construct validity. However, this was justified due to the lack of valid and reliable instruments to assess the knowledge of patients about Rheumatoid Arthritis. The study sample did not have a gender balance as Rheumatoid Arthritis is more frequent in women. Also, the psychometric properties have not yet been verified. Thus, we can suggest for future studies to verify intra- and inter- rater reliability of RAKAS-13/Brazil.

Given the importance of the knowledge of the disease to improve self-care and home-care in patients suffering from RA, mainly the elderly, we suggest the incorporation of RAKAS–13/BRAZIL to evaluate the knowledge of the disease. Beyond the identification of aspects related to the knowledge of patient about RA, it can contribute for modifications necessary to better self-management orientation in a patient-centered manner.

## Conclusion

The Brazilian Portuguese version of the RAKAS (RAKAS–13/BRAZIL) proved to be a questionnaire, that was easy and quick to administer to assess patient’s knowledge about RA despite its low correlation with PKQ in the present study.

## Electronic supplementary material

Below is the link to the electronic supplementary material.

**Additional file 1**: Changes in the process of translation and cross-cultural adaptation of RAKAS ?13/BRAZIL

**Additional file 2**: Changes in the process of translation and cross-cultural adaptation of RAKAS ?13/BRAZIL (back translation)

## Data Availability

All data generated or analysed during this study are included in this published article.

## Abbreviations

BMI: Body Mass Index
CHC: Complex Clinic’s Hospital of the Federal University of Paraná
CRP: C-reactive protein
DAS28: Disease Activity Score
ESR: Erythrocyte sedimentation rate
HAQ: Health Assessment Questionnaire
MMSE: Mini-Mental State Examination
PKQ: Brazil Patient Knowledge Questionnaire
RA: Rheumatoid Arthritis
RAKAS: Rheumatoid Arthritis Knowledge Assessment Scale
SD: Standard deviation
UFPR: Federal University of Paraná
