# Supplementary material for: Translation, transcultural adaptation into Brazilian Portuguese and concurrent validity of the rheumatoid arthritis assessment scale (RAKAS–13/Brazil)
Source: Adv Rheumatol. 2024 Jan 2;64:5. doi: 10.1186/s42358-023-00341-z (PMC13488765; doi:10.1186/s42358-023-00341-z)
Supplement: Supplementary file 1 [file 42358_2023_341_MOESM1_ESM.docx]

| **Additional file 1** Changes in the process of translation and cross-cultural adaptation of RAKAS –13/BRAZIL. | | | | | | |
| --- | --- | --- | --- | --- | --- | --- |
| **Original version** | | | **Brazilian Portuguese version (T1 – T2)** | | **Brazilian Portuguese version (T12)** | |
| The Rheumatoid Arthritis Knowledge Assessment Scale (RAKAS – 13) | | | T1: Escala de 13 itens sobre o Conhecimento de Artrite Reumatoide | | Escala de Avaliação de Conhecimento Sobre Artrite Reumatoide  (RAKAS –13/BRASIL) | |
|  |  |  | T2: Escala de Avaliação de Conhecimento sobre Artrite Reumatoide de 13 Itens (RAKAS – 13) | |  |  |
| **Item** | **Research Variable** | **Categories available** | **Variável de pesquisa** | **Categorias disponíveis** | **Variável de pesquisa** | **Categorias disponíveis** |
| 1 | *Do you know what rheumatoid arthritis is?* | *.Yes, completely aware*  *.Yes, to some extent*  *.No* | T1: Você sabe o que é artrite reumatoide? | .Sim, absolutamente  .Sim, um pouco.  .Não | Você sabe o que é artrite reumatoide? | .Sim, totalmente.  .Sim, um pouco.  .Não |
|  |  |  | T2: Você sabe o que é artrite reumatoide? | .Sim, plenamente ciente  .Sim, até certo ponto  .Não |  |  |
| 2 | *Which of the following is a symptom of rheumatoid arthritis?* | *.Low blood sugar*  *.Joint pain*  *.High blood pressure*  *.Feeling sleepy* | T1: Qual desses é um sintoma de artrite reumatoide? | .Açúcar baixo no sangue  .Dor na junta  .Pressão alta  .Sonolência | Quais desses é um sintoma da artrite reumatoide? | .Açúcar baixo no sangue.  .Dor nas juntas  .Pressão alta  .Sonolência |
|  |  |  | T2: Quais destes sintomas são de artrite reumatoide? | .Baixo açúcar no sangue  .Dor nas juntas  .Pressão alta  .Sonolência |  |  |
| 3 | *Which of the following is a risk factor of rheumatoid arthritis?* | *.High blood pressure*  *.High blood sugar*  *.Presence of diabetes in parentes*  *.Presence of rheumatoid arthritis in parents* | T1: Qual desses é um fator de risco para artrite reumatoide? | .Pressão alta  .Açúcar alto no sangue  .Parentes com diabetes  .Parentes com artrite reumatoide | Quais destes fatores são de risco para a artrite reumatoide? | .Pressão alta  .Açúcar alto  .Pais com diabetes .Pais com artrite reumatoide |
|  |  |  | T2: Quais destes fatores são de risco para a artrite reumatoide? | .Pressão alta  .Açúcar elevado no sangue  .Pais com diabetes  .Pais com artrite reumatoide |  |  |
| 4 | *Does rheumatoid arthritis only affects bones/joints?* | *.Yes*  *.No*  *.I don’t know* | T1: A artrite reumatoide envolve somente ossos/juntas? | .Sim  .Não  .Eu não sei | A artrite reumatoide atinge somente ossos e juntas? | .Sim  .Não  .Não sei |
|  |  |  | T2: A artrite reumatoide afeta somente ossos e juntas? | .Sim  .Não  .Não sei |  |  |
| 5 | *Can rheumatoid arthritis result in disability?* | *.Yes*  *.No*  *.I don’t know* | T1: A artrite reumatoide pode levar à incapacidade? | .Sim  .Não  .Eu não sei | A artrite reumatoide pode levar a incapacidade? | .Sim  .Não  .Não sei |
|  |  |  | T2: A artrite reumatoide pode ser incapacitante? | .Sim  .Não  .Não sei |  |  |
| 6 | *Can rheumatoid arthritis result in deformity?* | *.Yes*  *.No*  *.I don’t know* | T1: A artrite reumatoide pode levar à deformidade? | .Sim  .Não  .Eu não sei | A artrite reumatoide pode levar à deformidade? | .Sim  .Não  .Não sei |
|  |  |  | A artrite reumatoide pode causar deformidade? | .Sim  .Não  .Não sei |  |  |
| 7 | *Can rheumatoid arthritis spread from person to person?* | *.Yes*  *.No*  *.I don’t know* | T1: A artrite reumatoide pode passar de pessoa para pessoa? | .Sim  .Não  .Eu não sei | A artrite reumatoide pode passar de pessoa para pessoa? | .Sim  .Não  .Não sei |
|  |  |  | T2: A artrite reumatoide pode ser transmitida de uma pessoa a outra? | .Sim  .Não  .Não sei |  |  |
| 8 | *Does the disease run in family?* | *.Yes*  *.No*  *.I don’t know* | T1: Esta doença é hereditária? | .Sim  .Não  .Eu não sei | A artrite reumatoide é hereditária (de família)? | .Sim  .Não  .Não sei |
|  |  |  | T2: A artrite reumatoide é doença de família? | .Sim  .Não  .Não sei |  |  |
| 9 | *In terms of gender, who is more prone to suffer from this disease?* | *.Male*  *.Female*  *.Both have equal chance of suffering* | T1: Em termos de sexo, que tem mais artrite reumatoide? | .Masculino  .Feminino  .Os dois têm a mesma chance de ter | Em termos de sexo, quem tem mais chance de ter artrite reumatoide? | .Homem  .Mulher  .Os dois têm a mesma chance |
|  |  |  | T2: Em termos de gênero, quem é mais propenso a sofrer desta doença? | .Homens  .Mulheres  .Ambos têm a mesma chance de sofrer da doença |  |  |
| 10 | *Which of the following laboratory test is commonly used to assess this disease?* | *.ESR (Erythrocyte sedimentation rate*  *.Random Blood .Sugar*  *.Blood pressure*  *.Serum cholesterol* | T1: Qual dos seguintes exames de laboratório é mais usado para avaliar a doença? | .VHS (velocidade de hemossedimentação)  (glicemia)  .pressão arterial  .colesterol | Qual dos seguintes exames é mais usado para avaliar a doença? | .VHS (Velocidade de hemossedimentação)  .Glicemia  .Pressão arterial  .Colesterol |
|  |  |  | T2: Quais dos seguintes testes clínicos são usados comumente para avaliar a doença? | (Taxa de Sedimentação de Eritrócitos)  Teste Aleatório de Glicose  .Pressão sanguínea  .Nível de colesterol |  |  |
| 11 | *Is rheumatoid arthritis completely curable?* | *.Yes*  *.No*  *.I don’t know* | T1: A artrite reumatoide é completamente curável? | .Sim  .Não  .Eu não sei | A artrite reumatoide tem cura? | .Sim  .Não  .Não sei |
|  |  |  | T2: A artrite reumatoide tem cura? | .Sim  .Não  .Não sei |  |  |
| 12 | *Does it require lifelong treatment?* | *.Yes*  *.No*  *.I don’t know* | T1: Ela precisa de tratamento por toda a vida? | .Sim  .não  .Eu não sei | O tratamento da artrite reumatoide é por toda vida? | .Sim  .Não  .Não sei |
|  |  |  | T2: Exige tratamento vitalício? | .Sim  .Não  .Não sei |  |  |
| 13 | *Is physical therapy helpful in this disease?* | *.Yes*  *.No*  *.I don’t know* | T1: A fisioterapia é útil nesta doença? | .Sim  .não  .Eu não sei | A fisioterapia pode ajudar no tratamento da artrite reumatoide? | .Sim  .Não  .Não sei |
|  |  |  | T2: A fisioterapia é útil a esta doença? | .Sim  .Não  .Não sei |  |  |

T1: translation version 1; T2: translation version 2; T12: consensus version between T1 and T2.
