# Supplementary material for: Translation, transcultural adaptation into Brazilian Portuguese and concurrent validity of the rheumatoid arthritis assessment scale (RAKAS–13/Brazil)
Source: Adv Rheumatol. 2024 Jan 2;64:5. doi: 10.1186/s42358-023-00341-z (PMC13488765; doi:10.1186/s42358-023-00341-z)
Supplement: Supplementary file 2 [file 42358_2023_341_MOESM2_ESM.docx]

| **Additional file 2** Changes in the process of translation and cross-cultural adaptation of RAKAS –13/BRAZIL ( back translation). | | | | | | |
| --- | --- | --- | --- | --- | --- | --- |
| **Brazilian Portuguese version** | | | **Back-translation version after consensus meeting** | | **Back translated version (T12)** | |
| ESCALA DE AVALIAÇÃO DE CONHECIMENTO SOBRE ARTRITE REUMATOIDE (RAKAS –13/BRASIL) | | | T1: SCALE TO ASSESS KNOWLEDGE ABOUT RHEUMATOID ARTHRITIS | | SCALE TO ASSESS KNOWLEDGE ABOUT RHEUMATOID ARTHRITIS (RAKAS – 13) | |
|  |  |  | T2: SCALE TO ASSESS KNOWLEDGE ABOUT RHEUMATOID ARTHRITIS | |  |  |
| **Item** | **Variável de pesquisa** | **Categorias disponíveis** | **Research Variable** | **Categories available** | **Research Variable** | **Categories available** |
| 1 | Você sabe o que é artrite reumatoide? | .Sim, totalmente.  .Sim, um pouco.  .Não | T1: Do you know what rheumatoid arthritis is? | .Yes, completely.  .Yes, a little.  .No | Do you know what rheumatoid arthritis is? | .Yes, completely.  .Yes, a little.  .No |
|  |  |  | T2: Do you know what rheumatoid arthritis is? | .Yes, completely.  .Yes, a little.  .No |  |  |
| 2 | Quais desses é um sintoma da artrite reumatoide? | .Açúcar baixo no sangue.  .Dor nas juntas  .Pressão alta  .Sonolência | T1: Which of these is a symptom of rheumatoid arthritis? | .Low blood sugar.  .Joint pain  .High blood pressure  .Sleepiness | Which of these is a symptom of rheumatoid arthritis? | .Low blood sugar.  .Joint pain  .High blood pressure  .Sleepiness |
|  |  |  | T2: Which of these is a symptom of rheumatoid arthritis? | .Low blood sugar.  .Joint pain  .High blood pressure  .Sleepiness |  |  |
| 3 | Quais destes fatores são de risco para a artrite reumatoide? | .Pressão alta  .Açúcar alto  .Pais com diabetes .Pais com artrite reumatoide | T1: Which of these are risk factors for rheumatoid arthritis? | .High blood pressure  .High bloood sugar  .Parents with diabetes  .Parents with rheumatoid arthritis | Which of these is a risk factor for rheumatoid arthritis? | .High blood pressure  .High bloood sugar  .Parents with diabetes  .Parents with rheumatoid arthritis |
|  |  |  | T2: Which of these are risk factors for rheumatoid arthritis? | .High blood pressure  .High blood sugar  .One or both parents with diabetes  .One or both parents with rheumatoid arthritis |  |  |
| 4 | A artrite reumatoide atinge somente ossos e juntas? | .Sim  .Não  .Não sei | T1: Does rheumatoid arthritis affect only bones and joints? | .Yes  .No  .I don’t know | Does rheumatoid arthritis affect only bones and joints? | .Yes  .No  . don’t know |
|  |  |  | T2: Does rheumatoid arthritis affect only bones and joints? | .Yes  .No  .Don’t know |  |  |
| 5 | A artrite reumatoide pode levar a incapacidade? | .Sim  .Não  .Não sei | T1: Can rheumatoid arthritis lead to disability? | .Yes  .No  .I don’t know | Can rheumatoid arthritis cause disability? | .Yes  .No  .I don’t know |
|  |  |  | T2: Can rheumatoid arthritis lead to disability? | .Yes  .No  .Don’t know |  |  |
| 6 | A artrite reumatoide pode levar à deformidade? | .Sim  .Não  .Não sei | T1: Can rheumatoid arthritis lead to deformity? | .Yes  .No  .I don’t know | Is rheumatoid arthritis contagious? | .Yes  .No  .I don’t know |
|  |  |  | T2: Can rheumatoid arthritis lead to deformity? | .Yes  .No  .Don’t know |  |  |
| 7 | A artrite reumatoide pode passar de pessoa para pessoa? | .Sim  .Não  .Não sei | T1: Can rheumatoid arthritis spread from one person to another? | .Yes  .No  .I don’t know | Is rheumatoid arthritis contagious? | .Yes  .No  .I don’t know |
|  |  |  | T2: Is rheumatoid arthritis contagious? | .Yes  .No  .Don’t know |  |  |
| 8 | A artrite reumatoide é hereditária (de família)? | .Sim  .Não  .Não sei | T1: Is rheumatoid arthritis hereditary (in the family)? | .Yes  .No  .I don’t know | Is rheumatoid arthritis hereditary (in the family)? | .Yes  .No  .I don’t know |
|  |  |  | T2: Is rheumatoid arthritis hereditary (in the family)? | .Yes  .No  .Don’t know |  |  |
| 9 | Em termos de sexo, quem tem mais chance de ter artrite reumatoide? | .Homem  .Mulher  .Os dois têm a mesma chance | T1: Which sex has a greater chance of having rheumatoid arthritis? | .Men  .Women  .Both have the same chance | Which sex has a greater chance of having rheumatoid arthritis? | .Men  .Women  .Both have the same chance |
|  |  |  | T2: Which sex has a greater chance of having rheumatoid arthritis? | Men  Women  Both have the same chance |  |  |
| 10 | Qual dos seguintes exames é mais usado para avaliar a doença? | .VHS (Velocidade de hemossedimentação)  .Glicemia  .Pressão arterial  .Colesterol | T1: Which of these tests is most commonly used to assess the disease? | .ESR (Erythrocyte sedimentation rate)  .Glicemia  .Blood pressure  Cholesterol | Which of these tests is most commonly used to assess the disease? | .ESR (Erythrocyte sedimentation rate)  .Glycemia  .Blood pressure  Cholesterol |
|  |  |  | T2: Which of these tests is most commonly used to assess the disease? | .ESR (Erythrocyte sedimentation rate)  .Glycemia  .Blood pressure  Cholesterol |  |  |
| 11 | A artrite reumatoide tem cura? | .Sim  .Não  .Não sei | T1: Is there are a cure for rheumatoid arthritis? | .Yes  .No  .I don’t know | Is there a cure for rheumatoid arthritis? | .Yes  .No  .I don’t know |
|  |  |  | T2: Is there a cure for rheumatoid arthritis? | .Yes  .No  .Don’t know |  |  |
| 12 | O tratamento da artrite reumatoide é por toda vida? | .Sim  .Não  .Não sei | T1: Is treatment for rheumatoid arthritis lifelong? | .Yes  .No  .I don’t know | Does it require lifelong treatment? | .Yes  .No  .I don’t know |
|  |  |  | T2: Is treatment for rheumatoid arthritis lifelong? | .Yes  .No  .Don’t know |  |  |
| 13 | A fisioterapia pode ajudar no tratamento da artrite reumatoide? | .Sim  .Não  .Não sei | T1: Can physiotherapy help with rheumatoid arthritis? | .Yes  .No  .I don’t know | Can physiotherapy help with rheumatoid arthritis? | .Yes  .No  .I don’t know |
|  |  |  | T2: Can physiotherapy help with rheumatoid arthritis? | .Yes  .No  .Don’t know |  |  |

T1: translation version 1; T2: translation version 2; T12: consensus version between T1 and T2.
